# 

**Published:** 1896-10-24

**Authors:** 


**Notices of Births, Deaths, and Marriages** are inserted in this column at a charge of 2s. 6d. for an announcement not exceeding four lines.

### NOTICE TO CORRESPONDENTS.

All MS., Letters, Books for Review, and other matters intended for the Editor should be addressed The Editor, "The Hospital," The Hospital Building, 28 & 29, Southampton Street, Strand, W.C.

The Editor cannot undertake to return rejected MS., even when accompanied by stamped directed envelope.

### Notice to Advertisers and Subscribers.

All Advertisements, Orders for Copies of The Hospital, and Business Communications should be addressed to **THE MANAGER, (Not to The Eitdor),** The Hospital, The Hospital Building, 28 & 29, Southampton Street, Strand, London, W.C.

The Cost of Subscription, post free, is as follows:—Per week, 2½d.; per quarter, 2s. 8d.; per half-year, 5s. 3d.; per annum, 10s. 6d. Foreign:—Per annum, 15s. 2d.; payable, in all cases, in advance.

### IMPORTANT NOTICE.

The EDITORIAL and PUBLISHING OFFICES of "THE HOSPITAL" are now TRANSFERRED to their new premises, THE HOSPITAL BUILDING, 28 & 29, SOUTHAMPTON STREET, STRAND, LONDON, W.C.

**NOTICE.—Vol. XX. of "The Hospital" (April to September, 1896), in handsome cloth binding, is now on sale at the Office of this Journal, price 7s. 6d. Cases for binding the Numbers contained in this Volume 1s. 6d. Index to Vol. XX., 2½d., post free. The Monthly Part for November will be ready on November 2nd, Price 6d., by post 8d.**

### SCRAPS AND GLEANINGS.

Statistics prove that among every 35,500,000 railway travellers only one is killed.

A member of a medical society in Vienna has been expelled for criticising a fellow member in a daily paper.

During the month of July six patients were received at the St. George Hospital, Sydney, New South Wales.

It is proposed to erect a new infirmary at Colchester in place of the existing one, which is unsuitable and inadequate.

The cost of the erection of the West Highland Hospital, Oban, has been £2,600. This hospital is the gift of a Mrs. Parr.

A new operating theatre is needed at the Royal Cornwall Infirmary, Truro. It is calculated that the cost would be about £400.

The committee of the National Orthopædic Hospital have recently received from Miss Mary W. Harben the sum of £800 as a donation to secure a free bed in the institution.

The profit on the gala held recently on behalf of the Rochdale Infirmary was £180 on an expenditure of about £600. It is probable that another gala will be held next spring.

The musical festival held in the public park at Macclesfield on behalf of the General Infirmary has resulted in £29 10s. having been handed over to the authorities of that institution.

The suppression of street noises is being attempted in Milwaukee, U.S.A., by Health Commissioner Kempster. Chief among the noises which he thinks should be abolished is the church bell.

The United States sanitary inspector at Santiago de Cuba reports that yellow fever is still very prevalent and virulent in Cuba, and that the disease is causing many deaths among the soldiers.

The Mayor of Tunbridge Wells has kindly offered to defray the cost of completely installing the electric light throughout the General Hospital, which offer the governors have gratefully accepted.

The Cheshire County Council are busy with a scheme for the establishment of infectious diseases hospitals throughout the county, which is meeting on the whole with approval from the inhabitants.

About five hundred cases of typhoid fever are reported from Chicago, and in Denver (Colorado) an epidemic of typhoid fever is raging which has more victims and a greater fatality than any epidemic since 1892.

The Culham Rural District Council have secured two cottages on Clifton Heath for the purposes of an isolation hospital. Not more than two patients are to be received at one time, and one cottage is to be used for males and the other for females.

The splendid sum paid in in aid of the Wolverhampton and Staffordshire General Hospital as the result of the annual collections at the various industrial establishments throughout the district was £1,788, which is an increase of nearly £200 over last year.

During the past year 78 patients have been admitted to the Mirfield Memorial Cottage Hospital, a slight increase on the previous year; and the committee have the satisfaction of feeling that the hospital, which is now three years old, is doing very useful work in the district.

A hospital for nervous diseases is about to be opened in Belfast. A large house has already been engaged for the purpose, and a number of valuable instruments have been purchased. It is proposed to call the hospital the Queen Victoria Hospital for Nervous Diseases.

At a special meeting of ladies convened for the purpose of arranging scheme for the increase of the funds of the East Suffolk and Ipswich Hospital it was decided that a house-to-house canvass be undertaken by the ladies of Ipswich with the object of obtaining subscriptions, donations, and small contributions to this institution.

The recent cycle parade and fancy dress ball in aid of the Whitchurch, Salop, Cottage Hospital, despite the wet weather, produced a net result of £15. The prize fund was raised separately. The yeomanry and fire brigade headed the procession, which was a great success. Cyclists and machines were splendidly decorated and illuminated.

A resolution was passed at a meeting of the Hospital Chaplains' Union to the effect that the Hospital Chaplains' Union desire to express to Mrs. Benson and her family their sense of the great loss sustained by the whole Anglican Communion in the death of the Archbishop of Canterbury, and to place on record their appreciation of the deep interest he always showed in the welfare of the metropolitan and other hospitals.

The authorities at Salisbury evidently believe in the saying that "Continual dropping wears away a stone." In less than a month they have had three separate and distinct collections on behalf of the infirmary, viz., one of the local friendly societies, Hospital Sunday, and the infirmary anniversary, when a service was held at the cathedral in the afternoon, instead of the morning as hitherto, the former time being believed to be more convenient for the majority of people.

The site for the proposed infectious hospital for Rugby which has been chosen by the Urban District Council and sanctioned by the Local Government Board, is within the distance of a specified population prescribed for the building of a small-pox hospital, and under these circumstances the Rugby Council think it improbable they will be able to borrow the money for the erection; and it is their opinion that it would be useless to have the hospital unless it could be used for all infectious diseases.

The accommodation for cases of fever, diphtheria, &c., at Cardiff Sanatorium is very inadequate, and the Health Committee of Cardiff Corporation have decided that the present small-pox wards shall be used for cases of typhoid, diphtheria, &c., and that an isolated house be obtained in the event of any cases of small-pox arising. This is to cost £10,000, but it is doubtful whether even this additional accommodation will be adequate for a place such as Cardiff, with its large and increasing population and its influx of seafaring men from all quarters of the globe.

According to the report of Surgeon-Colonel Murata, of the Japan Red Cross Society, who was sent to Cuba by the Japanese Government to note the operations of the Spanish Army Medical Staff there, no such thing as disinfection of person or clothing seems to be known to the nurses in charge of the sick wards, and at an inspection of the wards that he witnessed by the surgeon-colonel in the Spanish Army, when seventy men, all suffering from wounds, were attended to, not once did the surgeon go through the form of disinfecting either his hands or his instruments.

### Books Received.

Witherby and Co.

"Lean's Royal Navy List, October, 1896."

John Wright and Co.

"Wright's Improved Visiting Lists, 1897."

Bailliere, Tindall, and Cox.

"Gout and Goutiness, and their Treatment." By William Ewart, M.D. Cantab.

"Guide to the Examination in Practical Chemistry for the Conjoint Board." By Percy A. E. Richards, F.I.C., F.C.S.

Saxon and Co.

"Everybody's Medical Guide." By M.D.Lond.

Bicker and Son.

"Agnes Jones." By Mrs. Roundell, with a supplement by Florence Nightingale and the Archbishop of Armagh.

**Periodicals and Pamphlets.**— Practitioner, Erin, Pediatrics, Reliquary and Illustrated Archæologist, Charlotte Medical Journal, Clinical Journal, Medical Week, Church of To-day, Westminster Review, Quarterly Medical Journal, Monthly Homœopathic Review, Nursing Record, Figaro, Invention, Medical Press, Medical Pioneer, New Saturday, Literary Digest, Electrical Review, Pears' Pictorial, London, Industries and Iron, Nursing Notes, Guy's Hospital Gazette, British and Colonial Druggist, The Sun, Columbus Medical Journal, Cassell's Family Magazine, The Planter, Medical Week, Electrical Engineer, St. Bartholomew's Hospital Journal.

### The Student of Medicine.

### APPOINTMENTS.

P. T. Adams, M.R.C.S., L.S.A., appointed Assistant Surgeon to Kent County Ophthalmic Hospital, Maidstone. S. H. B. Allison, M.B.,C.M.Edin., appointed Medical Officer for the Claudy Dispensary District of the Londonderry Union. M. Farrant, jun., M.R.C.S., L.R.C.P., appointed Medical Officer of Health to the St. Thomas Rural District Council and Medical Officer to the St. Thomas Union Workhouse. T. D. Forbes, M.B.,C.M.Edin., appointed Assistant Medical Officer to the Bethnal Green Union. J. H. Griffiths, M.B.,B.S.Lond., M.R.C.S., L.R.C.B., appointed Assistant Medical Officer to the Brook Hospital, Shooter's Hill, Kent. S. W. Haynes, M.D.,C.M.Edin., appointed Anæsthetist to the General Hospital, Birmingham. N. L. Hood, B.A.,M.B.,B.C.Cantab., appointed House Physician to the Hospital for Consumption and Diseases of the Chest, Brompton. F. C. W. Hounsell, B.A.Camb., M.R.C.S.Eng., appointed Medical Officer for the Bugbrooke District of the Northampton Union. C. H. Jackman, L.R.C.P. &S.Edin., appointed Medical Officer for the No. 10 District of the Hackney Union. W. G. Loveridge, L.R.C.P.&S., L.F.P.S.G., appointed Medical Officer of Health and Certifying Factory Surgeon for the Barton-on-Humber District. J. Meade, appointed Medical Officer for the Heworth District of the Gateshead Union. Dr. Roberts, appointed Medical Officer of Health to the Malling Rural District Council. W. J. Ruddock, M.B.Durh., M.R.C.S.Eng., appointed Medical Officer for the No. 5 District of the Newcastle Union. W. W. Shackleton, M.D.Dub., appointed Medical Officer for the Aldenham and Bushey District of the Watford Union. H. Sinigar, M.B., L.R.C.P., M.R.C.S., appointed Assistant Medical Officer to the St. Marylebone Infirmary. R. H. Weight, M.D.Edin., appointed Medical Officer for the Osbournby District of the Sleaford Union. W. R. White, M.D., appointed Medical Officer for the Benenden District of the Cranbrook Union. C. Whitehall-Cooke, M.D.Lond., M.R.C.S., L.R.C.P., appointed Surgeon to the Willesden Cottage Hospital.

### PASS LIST.

### University of Edinburgh.

Medical Preliminary Examination.

The following candidates have satisfied this examination: May Agnew, A. W. Atkinson, W. Black, R. J. Bradley, J. Brocket, Margaret E. Bryson, E. P. Calder, T. Cavanagh, Lizzie M. S. Clark, Paula Copeland, W. H. Dickinson, O. J. Evans, N. C. Fischer, E. C. Gimson, J. F. Halgert, D. C. Henry, E. M. Hingston, A. O. Hooper, Margaret D. Japp, W. Jarvis, E. Johnston, E. A. King, P. V. Langmore, G. J. Lecesne, W. H. Lucey, J. G. M'Bride, H. K. Macdonald, W. J. Macdonald, D. W. Macfarlan, J. P. M'Farlane, A. P. Gregor, J. M'Kenzie, Clara V. M'Laren, J. M'Leod, G. M'Neill, K. D. C. Macrae, J. G. Millar, C. E. S. Mitchell, R. S. Munro, A. D. Murray, J. Neil, P. Neuman, T. F. Norbury, W. Ottewill, Sophie Palmer, J. N. D. Paulson, W. D. Pierce, J. J. N. Pierre, H. St. J. Randell, R. Rorie, G. H. Skinner, W. B. Tannahill, P. Telles, T. L. Thomson, P. S. Tillard, G. C. Trotter, C. E. Watts, E. Wells, C. E. Wilson, F. E. Wilson, H. S. Winterbotham, and D. Young.

### The Royal University of Ireland.

First Examination in Medicine.

The following candidates have satisfied the examiners: A. L. Black, Queen's College, Belfast; J. S. Cargin, Queen's College, Belfast; J. Carroll, University College, Dublin; R. H. Caughey, Queen's College, Belfast; R. Cogan, Queen's College, Cork; W. Donnan, Queen's College, Belfast; J. McC. Gibson, Queen's College, Belfast; J. J. Gillis, Queen's College, Belfast; J. T. Grehan, University College, Dublin; O. E. Jackson, Queen's College, Belfast; J. I. Jaffé, Queen's College, Cork; J. C. McCarroll, Queen's College, Belfast; J. E. MacIlwaine, Queen's College, Belfast; A. Riddell, Queen's College, Belfast; H. Ross, Queen's College, Cork; H. E. Rutherford, Queen's College, Belfast; E. F. Scott, Queen's College, Galway; M. A. Shinkwin, Queen's College, Cork; J. Stewart, Queen's College, Belfast; W. A. Stoops, Queen's College, Belfast; W. J. Thompson, Queen's College, Belfast.

The following candidates may present themselves for the further examination for honours in the subjects set opposite their names: A. L. Black, botany, zoology, experimental physics; J. Carroll, botany, chemistry, experimental physics; R. H. Caughey, chemistry and experimental physics; J. J. Gillis, botany; O. E. Jackson, chemistry; J. I. Jaffé, botany; J. E. McCarroll, experimental physics; J. E. MacIlwaine, botany, zoology, chemistry, experimental physics; H. Ross, botany; H. E. Rutherford, botany and zoology; M. A. Skinkwin, botany; J. Stewart, botany; W. A. Stoops, zoology and experimental physics; W. J. Thompson, botany, zoology.—Note: Candidates recommended in any two subjects will be allowed to present themselves at the further examination in all subjects.

### St. Thomas's Hospital.

Entrance Scholarships in Natural Science.—First (£150) awarded to Alfred Barton Lindsey; second (£60), awarded to Robert Ellis Roberts. Messrs. C. Newton Sears and W. H. Harwood-Yarred obtained marks qualifying for a scholarship.

Entrance Scholarship for University Students, value £50, awarded to Mr. Raymond J. Horton-Smith, B.A., of St. John's College, Cambridge.

